# There Is No Dilemma: The Issue of Female Genital Reinfibulation in High‐Income Countries Need Not be Revisited

**DOI:** 10.1111/jep.70303

**Published:** 2025-10-18

**Authors:** Richard C. Armitage

**Affiliations:** ^1^ Academic Unit of Population and Lifespan Sciences, School of Medicine University of Nottingham Nottingham UK

According to the World Health Organisation, female genital mutilation (FGM) comprises all procedures that involve the partial or total removal of external female genitalia or other injury to the female genital organs for nonmedical reasons [1]. The same classification describes Type III FGM—often referred to as infibulation—as the narrowing of the vaginal orifice through the creation of a covering seal formed by cutting and apposition of the labia minora and/or the labia majora, with or without excision of the clitoris [1].

Defibulation is the anterior midline vulval incision of the infibulation scar performed prenatally or at the time of delivery. While it restores sufficient access to the female reproductive tract, defibulation does not reinstate physical or emotional normality [2]. Reinfibulation is the subsequent resuturing of the incised infibulation scar tissue after delivery [2]. Approximately 13 million women have undergone infibulation globally, and an estimated 6.5–10.4 million women are likely to have been reinfibulated worldwide [2].

The prevalence of FGM is highly concentrated in a band of countries from the Atlantic coast to the Horn of Africa [3], and reinfibulation is most prevalent in countries where Type III FGM is the most practiced type, including Somalia (98%–100%), Sudan (82%), Djibouti (50%), and Eritrea (34%) [1]. While offering no medical benefits, reinfibulation returns women who have been defibulated to an infibulated state and subjects them once again to its accompanying harms. The process is associated with, among other risks, localised infection or abscess formation, sepsis, haemorrhage, shock, acute urinary retention, and contraction of hepatitis and/or HIV [4], while the infibulated state is associated with a plethora of gynaecological and obstetric complications [4, 5].

In their recent Correspondence in *The Lancet*, Bonavina and colleagues lament the unlawful status of reinfibulation in high‐income countries as ‘potentially depriving [women] of their choice’, and claim there exists an ethical dilemma between preserving the ability of women to choose reinfibulation and protecting them against its harmful consequences [6]. The article contains multiple ethical problems, which I addressed in a reply submitted to the *The Lancet*. The reply was rejected by the editorial board, and my subsequent request for the board′s reasoning behind its decision was not granted. I shall, therefore, rebut the article here instead.

## 1 A Flawed Conception of Autonomy

In their article, Bonavina and colleagues presume that the women who ‘choose’ reinfibulation simply exercise their autonomy in doing so. This reflects a superficial understanding of autonomy that conflates mere expression of preference with genuine autonomous choice. Autonomy, properly conceived, requires not only the capacity to express preferences, but the necessary conditions for authentic independent self‐determination [7], specifically the absence of undue pressure, solicitation or coercion [8].

In addition to this voluntariness, true autonomy requires adequate information pertaining to the decision and its consequences, and the cognitive ability to understand it [8]. Furthermore, relational accounts of autonomy argue that meaningful autonomy requires supportive social conditions, such as recognition, care, and non‐oppressive relationships, that enable individuals to develop the necessary psychological and social capacities for authentic self‐determination [9]. When examining a woman's ‘choice’ for reinfibulation, each of these conditions is likely to be compromised.

First, the informational requirements for autonomous choice are rarely met in this context. Women who undergo reinfibulation often lack comprehensive understanding of the associated medical risks, especially since the victims of FGM are nearly always minors [10]. Further, the ‘choice’ frequently occurs within informational environments dominated by traditional narratives that present reinfibulation as normal, necessary, or inevitable [10].

Second, it is far from clear that the women who ‘choose’ reinfibulation do so with the required voluntariness and freedom to choose otherwise (again, especially since the victims of FGM are nearly always minors) [10], including in the high‐income countries into which they, their cultural practices, and social norms migrate [10]. Rather, the voluntariness requirement is fundamentally compromised by the coercive social structures within which these ‘choices’ occur. Notably, Bonavina and colleagues themselves acknowledge the pressures of community members, specifically ‘older female counterparts’, on ‘the decision to perform reinfibulation, influenced by the conviction that the procedure will improve male sexual pleasure.’ [6].

## 2 The Limits of Autonomy in Medical Practice

Even if we were to grant that a woman's request for reinfibulation could be autonomous, this would not establish a medical duty to perform the procedure. This is because autonomy is only one of multiple ethical principles that often lie in tension with one another, most notably of which in this context is non‐maleficence, [7, 8] which warns against procedures that cause significant harm without corresponding medical benefit.

For example, a prospective kidney donor's autonomous decision can be ethically overruled due to an unacceptable surgical risk, while a doctor is under no ethical obligation to honour my autonomous request to remove my healthy right arm. Moreover, doctors cannot be required to perform actions that they deem to be unethical and that would violate their integrity if they did so, even when patients autonomously request them [11]. Ethical principles are to be balanced and none, including autonomy, are sacrosanct. The extreme harm caused by reinfibulation place it far beyond the boundaries of ethical practice, autonomy notwithstanding.

## 3 Further Problems: Cultural Relativism and Human Right Violations

Bonavina and colleagues appear to mount a cultural relativist argument by suggesting that prohibitions on reinfibulation represent cultural imperialism (‘no reinfibulation policies might be seen as a mechanism by which countries reinforce their culture, ideologies, and values as instruments of power for social order, and being the default standard for global human rights.’) [6]. This argument fails to distinguish between tolerance of cultural diversity and tolerance of harmful practices. Cultural practices that systematically harm and subordinate women cannot claim moral protection merely by virtue of their traditional status [12].

Further, the human rights framework endorsed by virtually all high‐income countries establishes universal standards for bodily integrity and gender equality that transcend cultural boundaries [13]. These standards recognise that some practices, regardless of their cultural provenance, are incompatible with human dignity and cannot be sustained through appeals to cultural authenticity. Infibulation, and all other forms of FGM, fall squarely into this category.

## 4 Conclusion

Accordingly, the apparent ethical dilemma identified by Bonavina and colleagues simply does not exist. To the contrary, reinfibulation, like all forms of FGM, is ethically indefensible, and ‘no reinfibulation’ policies in high‐income countries must be upheld to safeguard vulnerable women. While the authors see such policies as ‘a mechanism by which countries reinforce their culture, ideologies, and values’, [6] the element of any country's culture, ideology, and values that rejects such barbarism is ethically commendable.

## Conflicts of Interest

The author declares no conflicts of interest.

## Data Availability

The author has nothing to report.
